# FTO rs 9939609 SNP Is Associated With Adiponectin and Leptin Levels and the Risk of Obesity in a Cohort of Romanian Children Population

**DOI:** 10.1097/MD.0000000000003709

**Published:** 2016-05-20

**Authors:** Carmen Duicu, Cristina Oana Mărginean, Septimiu Voidăzan, Florin Tripon, Claudia Bănescu

**Affiliations:** From the 1st Department of Pediatrics (CD, COM); Department of Epidemiology (SV); and Department of Genetics (FT, CB) and CCAMF (CB) University of Medicine and Pharmacy Tîrgu Mureş, Romania.

## Abstract

Obesity is a disorder with increasing frequency in children and adolescents, directly linked with various diseases. Variants in the *FTO* (fat mass and obesity-related) gene have been associated with body mass index and waist and hip circumferences in widespread populations.

The aim of this case-control study was to assess if there is any association between *FTO* gene variants rs9939609, respectively, rs17817449 with anthropometric and metabolic biomarkers (fasting glucose, TC, HDL-cholesterol, LDL-cholesterol, triglycerides) and adipokines (adiponectin and leptin), in Romanian obese children.

A total of 387 children, 201 obese and 186 nonobese individuals, were included in this prospective study. Genotyping of the *FTO* gene polymorphisms for all subjects was performed using the restriction fragment length polymorphism (PCR–RFLP) method.

Significant associations were found between *FTO* rs9939609 single nucleotide polymorphism (SNP) and obesity. AA genotype carriers have a 2.02 times higher risk for obesity compared with AT+TT genotype carriers. Risk allele carriers of rs17817449 SNP had somewhat higher values of weight, body mass index, waist and hip circumference, total cholesterol, triglycerides, adiponectin, and fasting glucose.

This study revealed the genetic association between rs9939609 SNP of *FTO* and obesity in a Romanian population, and to the authors’ knowledge, this is the first study to investigate this association in a Romanian population. This study also established that combined variant genotypes (AA/GG) of *FTO* rs9939609 /rs17817449 are strongly associated with several measures of adiposity (weight, BMI-SD, mid-upper arm circumference, tricipital skinfold thicknesses) and are also associated with total cholesterol, triglyceride, and LDL-cholesterol levels.

## INTRODUCTION

One of the major public health problems of our century in children and teenagers is obesity, whose incidence has increased considerably during the last 3 decades.^1^ Obesity is a multifactorial disorder, and its etiology is influenced by genetic background as well as by environmental factors.^2^

Epidemic proportions of childhood obesity have also been observed in Romania.^3–5^

One of the genes that has been strongly and consistently found to be associated with common obesity is *FTO* (fat mass and obesity-related) gene.^2^ Current data provide more reliability to the theory that obesity may be an inherited neurobehavioral disorder through the role of *FTO*, which is implicated in the mediated control of food intake.^6,7^ The effect of *FTO* on body composition and consequently on the risk of obesity and overweight was observed also in children.^8,9^ Some researchers found in their works that the *FTO* gene and its SNPs (single nucleotide polymorphisms) stand for a significant predictor of BMI.^8–10^ The *FTO* gene variants are associated with BMI (body mass index) in Caucasian children and adults, as well as other populations: Asians, Africans, and Americans.^8,9,11–15^

In recent years, a small number of studies noticed the role of a group of common SNPs of *FTO* with regard to obesity,^8,9,16–20^ but there are still many unknowns regarding their function.^17^

The link between the *FTO* gene polymorphism and obesity has been confirmed in several populations,^8,9,21–26^ although no data on Romanian children are available.

Because of the high incidence of childhood obesity in our country, we tested the association of the *FTO* rs9939609 and rs17817449 SNP with some obesity-related anthropometric and metabolic parameters in a large children cohort.

## MATERIALS AND METHODS

The present study included a total of 387 cases aged between 1 and 18 years. The study groups comprised 201 Romanian overweight and obese children and 186 normal-weight participants, evaluated in Pediatric Department between 2012 and 2015. The control group included apparently healthy children, which were investigated for minor healthy problems (constipation, lower urinary tract infection, viral respiratory tract infection, etc), without any chronic disease that could influence the results.

### Physical Examination and Anthropometric Characteristics

A standardized physical exam was performed in all children. Anthropometric evaluation included: body weight (kg), height (cm), waist circumference, hip circumference, mid-upper arm circumference (MUAC), and tricipital skinfold thicknesses (TST). The formula: body weight (kg) divided by the square of the height (m) was used to assess BMI. All measurements were done on the right side of the subject and were obtained in triplicate by a team of trained persons. Body weight was measured with a daily calibrated scale. To evaluate height, a daily calibrated pedometer was used. MUAC was measured midway between the apex of the shoulder and elbow with the use of a measuring tape, whereas TST was evaluated in the posterior upper arm making use of a thickness calliper. In standing position, we assessed waist circumference at the midpoint between the iliac crest and the lower costal margin and hip circumference at the greatest circumference over the buttocks. The anthropometric values were converted to SD for age and sex with the use of the Switzerland Growth Chart (1989) curves^27^; the physiologic data reference values were between − 2.0 and + 2.0 SD. We considered obesity for children who had anthropometric parameters > 2.0 SD. To evaluate obesity and overweight status among our patients, we used the WHO Z score of BMI for age. BMI standard deviations above + 1 for overweight, between + 2.0 and  + 3.0 SD for moderate obesity and above + 3 for severe obesity were used.^28,29^ The metabolic laboratory tests evaluated in all cases were: total protein and albumin, cholesterol, triglycerides, LDL-cholesterol (low-density lipoprotein), HDL-cholesterol (high-density lipoprotein), adipokines like adiponectin, and leptin.

### Genotyping

Genomic DNA was isolated from EDTA-anticoagulated whole blood samples using Quick-gDNA MiniPrep kits (ZymoResearch, USA) according to the manufacturer's protocol and stored at –20 °C. For genotyping of SNP variant rs9939609, we used a PCR–RFLP assay as described by López-Bermejo et al.^30^ Genotyping of the SNP variant rs17817449 was performed by the PCR–RFLP assay as previously described.^31^

### Statistical Analysis

The frequencies of alleles and genotypes were determined by counting the genotypes. Distribution of genotypes in both groups was compared by chi-square or Fisher exact test. The studied variables were described as frequencies and percentages or as means and SD; inferential statistics was done using the nonpaired Student's *t* test for quantitative variables and the Fisher exact test for qualitative variables. Nonparametric Kruskal–Wallis and Mann–Whitney tests were used to compare 3 or more groups. Statistical tests were performed using the SPSS v 18.0 software. Odds ratios (OR) with 95% confidence intervals (CI) were calculated to estimate the association between geno types and measurements. A *P* value < 0.05 was considered to have a statistical significance.

### Ethics

The children's parents or guardians gave their written informed consent prior to inclusion in the study. The Ethics Committee of the University of Medicine and Pharmacy of Tirgu Mures approved the study No 13/18 July 2011, which conforms to the guidelines of the Declaration of Helsinki.

## RESULTS

Genotyping was performed for 2 *FTO* SNPs (rs9939609 and rs17817449) on 387 samples. The study group was composed of 45.74% boys (n = 177) with mean age of 9.51 years and 54.26% girls (n = 210) with mean age of 10.69 years. Clinical features of the participants in this study are shown in Table 1. The median BMI was 22.3 kg/m^2^.

**TABLE 1:**
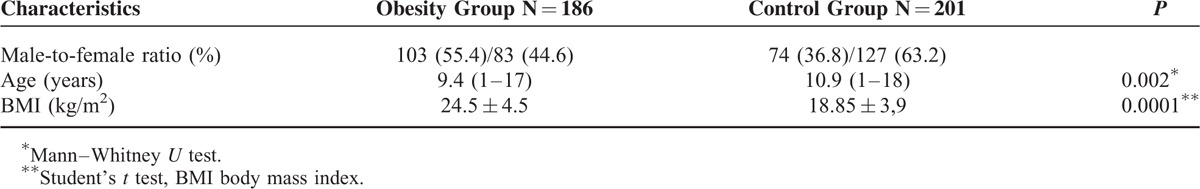
Main Characteristics of the Study Participants

Boys had a higher median BMI (23.75 kg/m^2^) than girls (21.50 kg/m^2^), *P* = 0.001. Anthropometric and laboratory parameters of the obese children according to their genotype for each *FTO* SNP are shown in Table 2.

**TABLE 2:**
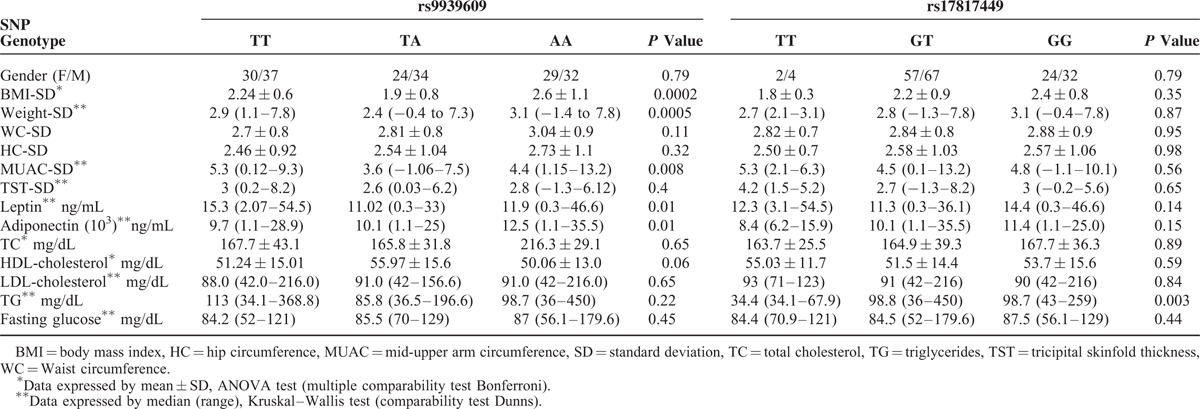
Clinical Parameters and the rs9939609 and rs17817449 *FTO* Gene Polymorphisms Within the Obese Group

### FTO rs9939609 (T–normal Allele; A–variant Allele)

Compared to obese children, the proportion of the AT genotype carriers was significantly higher in normal-weight controls (*P* = 0.04), whereas the proportion of homozygous AA obese children was significantly higher than controls (*P* = 0.003). Wild-type genotypes (TT) were approximately equally distributed within the groups.

Obese homozygous AA carriers showed significantly elevated values for weight-SD and BMI-SD. Higher measures of waist and hip circumference were observed in children carrying the A allele than in those who did not have the risk allele, but without statistical difference.

A trend of elevated values of anthropometric parameters (BMI-SD, weight-SD, MUAC-SD, and TST-SD) was observed in homozygous TT and AA carriers when analyzed separately in the obese and control group, but without significant difference. A significant difference of MUAC-SD was achieved in wild-type (TT) homozygotes compared to heterozygotes. Furthermore, no significant elevations were found in total cholesterol, LDL-cholesterol, HDL-cholesterol, triglycerides, fasting glucose, neither in the homozygous nor in the heterozygous cases. A significantly higher level of LDL-cholesterol was noticed in obese AA homozygotes compared to control AA homozygotes (*P* = 0.03).

For adipokines (leptin, adiponectin), a significant difference was found for leptin in wild-type (TT) carriers, whereas significantly higher levels of adiponectin were found in homozygous AA carriers. When we used the additive model (AA + AT), A allele carriers had significantly higher values for adiponectin (*P* = 0.03) only. In the control group, we did not observe any association between genotypes and anthropometric as well as metabolic parameters (data not shown).

**FIGURE 1:**
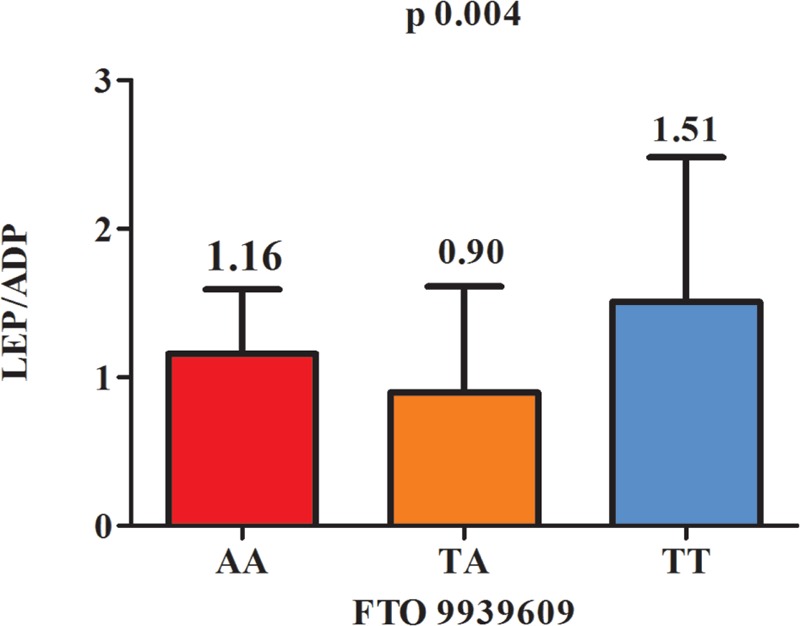
The median values of the leptin/adiponectin ratio according to the FTO 9939609 genotypes. ADP = adiponectin, LEP = leptin.

**FIGURE 2:**
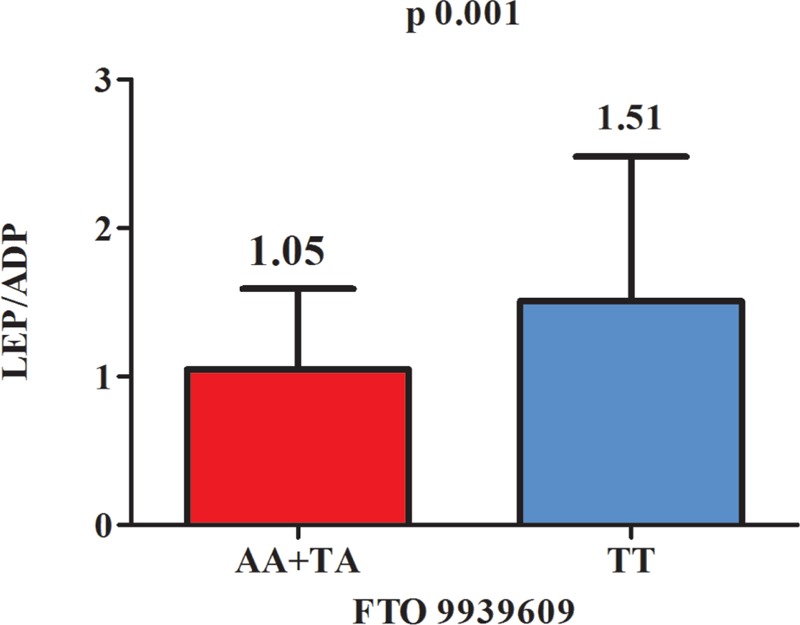
The median values of leptin/adiponectin ratio for variant alelle carriers compared to homozygous normal alelle carriers FTO 9939609. ADP = adiponectin, LEP = leptin.

The median value of leptin/adiponectin ratio was 1.29 with a minimum of 0.02 and a maximum of 16.15, whereas the median value for the adiponectin/leptin ratio was 0.775 with the minimum of 0.06 and the maximum of 46.32. When we used the leptin/adiponectin ratio a significant statistical difference among FTO 9939609 genotypes was found (*P* = 0.004) (AT vs TT) (Figure 1). Using this ratio, a significant difference was remarked among A allele carrier (AA + AT) compared to T allele homozygotes (Figure 2). Moreover, we found a significant positive correlation between the leptin/adiponectin ratio and BMI just/merely in AT genotypes FTO 9939609. Regarding the rs17817449 FTO gene no remarkable difference was noticed.

No association was found between A allele carriers and BMI-SD in the obese group nor in the control group (0.18 and 0.41, respectively; Mann–Whitney test). We observed that AA carriers have a 2.02 times risk for obesity compared with AT + TT carriers (OR 2.027, 95%CI 1.274–3.226, *P* = 0.002). We did not observe a significant difference regarding BMI-SD in obese AA carries after adjusting for gender. When we performed gender-stratified analysis, we observed that FTO genotype had no influence on BMI in males or females.

In applying Kruskal–Wallis and Dunns tests, we looked for a relationship between A allele carriers and the severity of obesity in accordance with age, but no association was found within the 2 groups.

When we used the additive model (AA + AT), we did not notice any difference among the 2 study groups.

After a logistic regression test was applied, we observed a significant association between A allele carriers and BMI-SD in children >9 years, A allele being a risk factor for a higher BMI (OR 4.93, 95%CI 2.126–11.4756, *P* = 0.0002).

Using Mann–Whitney nonparametric test, we observed that obese A allele carriers aged 5 to 9 years had higher BMI-SD (*P* = 0.72), whereas in the control group risk (A), allele carriers had significantly lower BMI-SD than children without this allele (*P* = 0.002). In the obese group, we noticed that AA homozygotes had higher values for weight, BMI, and waist and hip circumferences compared with TT homozygous.

### FTO rs17817449 (T—normal Allele, G—variant Allele)

The TT homozygous was found just in the obese group. The allele frequency was comparable between the obese and control groups. The obese G allele carriers (GG + GT) had significantly higher values of triglycerides comparing with the TT homozygous. Also, higher values of triglycerides were noticed in the G allele carriers in the control group, but without significant difference. No association was achieved between the genotype and the severity of obesity after adjustment for age and sex.

In the obese group, G allele carriers had a slightly higher BMI-SD than T allele carriers (*P* = 0.42). After gender adjusting GG homozygous had higher waist circumference (*P*>0.05).

Slightly higher measures of waist and hip circumferences were observed in children carrying the G allele compared to children who did not have the risk allele. Also, we did not find any association between waist as well as hip circumferences and genotype according to gender.

The triglyceride serum level was found to be higher in risk allele carriers (GG + GT) than in the homozygous T allele group (*P* = 0.001). Adiponectin levels in subjects with TT genotype were lower than in subjects with GG or GT genotype (*P* = 0.63). We also found marginally increased levels of fasting glucose in GG and GT genotype subjects (*P* > 0.05).

When we used the additive model rs9939609/rs17817449 AA/GG, we observed that weight-SD, BMI-SD, MUAC-SD, TST-SD, total cholesterol, triglyceride, and LDL-cholesterol were significantly higher than in the control group. When we used other additive models for the 2 investigated SNPs (GG/AT; GG/TT; GT/AT; GT/TT), significant differences were also observed, but for all parameters (BMI-SD, MUAC-SD, TST-SD).

Children carrying the G/A haplotype (rs17817449/rs9939609) did not have a higher risk of obesity (OR 0.86, 95%CI 0.57–1.31, *P* = 0.50) compared to those with the reference T/A haplotype.

## DISCUSSIONS

In this article, we analyzed the single nucleotide polymorphisms rs9939609 and rs17817449 of the *FTO* gene in a group of obese and normal-weight Romanian children.

According to the recent studies,^3–5^ the prevalence of obesity in Romanian children is high similar with the obesity rate observed in Central and Eastern Europe (8%).

A strong connection between 2 SNPs of the *FTO* gene (namely rs9939609 and rs17817449), and BMI has been reported by several studies even in children.^8,9,26^ On the other hand, a recent study did not find any association between the *FTO* gene and obesity.^32^

The strongest effect seen on adiposity was in relation with SNP rs9939609,^8,9,16,26^ whereas the study realized by Lombard et al on South African adolescents showed a mild association among the *FTO* SNP rs17817449 and BMI after age, gender, and gender-specific pubertal stage adjustment.^7^ A very recent meta-analysis highlighted the strong association between the *FTO* gene and adiposity in childhood and adolescence.^25^

The first linkage between 9939609 variant allele with BMI was reported in a genome-wide association study,^9^ the same conclusions being replicated later in other studies.^18,20^ The same conclusion was highlighted by a recent meta-analysis of genome-wide association studies.^33^ Other studies have observed comparable associations for the rs8050136 A allele in Caucasians.^18,20^ Higher BMI and waist circumference values have also been observed in Europeans in strong association with the variant alleles *FTO* rs17817449 and rs1421085.^34^ Some data sustain the fact that *FTO* polymorphisms may exert a greater effect along with growing-up. This was consistent with the results of a longitudinal study that confirmed a stronger linkage among BMI and the FTO rs9939609 A allele in children aged 11 years,^35^ close to our results, where we found this association in children >9 years. In another study, the association between *FTO* rs9939609 SNP and obesity was seen in children from 7 to 9 years old, in whom A allele was linked with body weight as well as fat mass.^9^

A higher BMI-SD in rs9939609 A allele carriers was found in our study group, but without significant association (median BMI-SD 2.14, *P* = 0.18). A significant positive association between the rs9939609 A allele and higher BMI was found by Grunnet et al.^36^

In the study conducted by Mangge et al, the proportion of *FTO* rs9939609 AA genotype was significantly higher in the obese adolescents compared to the controls.^37^ Heterozygous and wild-type genotypes were approximately equally distributed within the normal weight and obese adolescents.^37^ The observations made by Mangge et al in juveniles are in accordance with our results and with the observations of adults by Frayling et al.^9^ Similar results were observed in our study, where the AA genotype was 2 times more frequent in the obese group compared with the control group.

We found positive association of the SNP rs9939609 with BMI-SD and the risk of obesity (OR 1.54, 95%CI 0.91–2.60, *P* > 0.05) for the AA homozygotes. After age adjustment, we observed that A allele was related with a higher BMI-SD in children >9 years that conferred an increased risk of obesity (OR 4.93, 95%CI 2.126–11.4756, *P* = 0.0002), whereas Frayling et al found this risk factor in children >7 years.^9^

In our study, AA homozygotes showed significantly elevated values for BMI-SD and waist circumference-SD, whereas Mangge et al^37^ observed that AA homozygotes had significantly elevated values for body weight, BMI, and waist and hip circumferences.

In another study, children with AA genotype had a significantly higher BMI-SD,^11^ similar to our results. These differences were seen in boys (*P > *0.05) compared to girls and are similar with another study.^11^ In contrast to our findings, this association has been confirmed in girls but not in boys.^38^ In a Dutch study, the A allele was present in 88% of the overweight and obese children compared to the lean children in whom A allele was present in 45%.^39^ These results are in discrepancy with ours as we found approximately similar percentage within the 2 groups, but similar to those in obese Polish children (48.4 versus 49%).^11^ The association of the *FTO* rs9939609 A allele with a higher frequency of overweight and obesity coupled with an increased BMI as well as central body fat distribution was found out by a recent study.^40^

The findings regarding the association and the risk for obesity conferred by *FTO* rs9939609 gene polymorphism was comparable with the data reported by different association studies in children,^8,9^ therefore confirming this as an obesity susceptibility gene in children.^40^ Other research found a strong association of SNP rs17817449 with obesity,^41^ association that was not replicated in our study.

In our research the mean values for BMI were 24.29, 23.55, and 25.77 for AA, AT, and TT genotypes which are different from those obtained by Xi et al in their study (23.0, 22.5 and 21.7).^42^ The explanation of these discrepancies could be the small number of cases and the ethnicity. In accordance to the results of other studies,^9,43^ we found no significant associations between the *FTO* polymorphisms and body weight and length, respectively.

Rutters et al found that the *FTO* A allele was associated with higher BMI and leptin levels at the age of 12, association that became stronger at the age of 17.^39^ This association was not observed in our study, a fact that could be explained by our smaller number of cases.

Labayen et al have shown that there is a significant correlation between the minor A allele of the *FTO* rs9939609 polymorphism and elevated serum leptin levels in adolescents,^44^ this finding being in contradiction to our results. A recent study found no association between *FTO* rs9939609 and metabolic biomarkers (fasting glucose, TC, HDL-cholesterol, LDL-cholesterol, triglycerides) and adipokines (adiponectin and leptin), results partially overlapping our observations.^37^ Univariate analysis revealed no associations between the *FTO* 9939609 gene variants and the levels of total cholesterol, triglycerides, and fasting glucose, whereas other studies did.^11^

We also observed an association between the A allele of the *FTO* rs9939609 gene and an increased BMI as it was established in other study.^45^ Somewhat higher measures of waist and hip circumferences were noticed among the A allele carriers of the *FTO* rs9939609 compared with children who did not have the variant allele, but without significant differences, similar with those reported in another research,^46^ this differentiation being significant in other studies.^37,40^

The influence of *FTO* rs17817449 polymorphism on obesity was investigated by Scott et al in children.^31^ They reported that this polymorphism might have an effect on variation in obesity-associated measures in teenagers and that homozygous for G allele have higher levels of adiposity compared to T allele carriers, similar to our results. Contrarily in our study, we did not observe a significant gender interaction with BMI.

In their research, Lombard et al found the minor allele of rs17817449 *FTO* gene to be associated with higher BMI values.^7^ In their study, Do et al found significant associations of rs17817449 with BMI, weight, and waist circumference, demonstrating its involvement in the etiology of obesity, insulin resistance, and increased plasma leptin levels.^34^ In disagreement with these studies, no significant associations between the *FTO* polymorphism and body weight, BMI, waist circumference, and plasma leptin levels were found in the present research. Higher values for waist circumference and hip circumference were seen in G allele carriers.

According to Mente et al, there is a positive link between leptin levels and obesity and insulin resistence.^47^ It is already well-known that there is an opposite relation of adipokines with fat mass, a direct relation of leptin, and a reverse relation of adiponectin, respectively.^48^ Our results show higher leptin levels in obese children; these increase being significant in normal allele carriers irrespective of FTO SNP. In some results from the literature, the leptin/adiponectin ratio express a more intrinsic role in related inflammatory process on obesity than these hormone observed alone in its concentrations.^47–51^ In our research, we found a significant correlation of leptin/adiponectin ratio just with BMI only in FTO rs9939609 AT genotype. No correlation of leptin/adiponectin ratio was found with the others antropometric data (MUAC, TST, waist circumference, hip circumference). These findings are partially overlapping with those from other studies.^47,48^ Similar to our results, recent pediatric studies documented a higher leptin/adiponectin ratio in the overweight and obese children than in nonobese children, which correlated positively with BMI or abdominal fat mass.^49–51^

Moreover, the leptin/adiponectin ratio was higher in obese children, and a stronger association with BMI than adiponectin alone was find out. Previous studies established that leptin levels are increasing in girls and decreasing in boys with advancing/growing age and because of puberty hormonal changes.^52,53^ However, our study did not confirm any age-related increase in leptin levels in girls.

The strength is represented by the fact that this is a pilot study that presents for the first time evidence about the role of genetic variants with regard to obesity risk in a Romanian children population and the weaknesses of our study consist of investigation of only 2 SNPs of the *FTO* gene and the moderate power in investigating the association with obesity as not all FTO polymorphisms were investigated, there were only 387 cases and it was a single-center study.

Additional prospective studies that appraise the influence of these *FTO* polymorphisms in childhood obesity are needed to verify our findings.

## CONCLUSION

Our findings showed that rs9939609 SNP was significantly associated with obesity in our sample, whereas this association could not be replicated in the rs17817449 SNP. This study provides the first evidence about the association of *FTO* rs9939609/rs17817449 AA/GG with obesity in this population. Our data attest the previously reported association regarding the genetic variability in intron 1 of *FTO* gene and obesity risk.
